# MicroRNA-24-3p regulates neuronal differentiation by controlling hippocalcin expression

**DOI:** 10.1007/s00018-019-03290-3

**Published:** 2019-09-05

**Authors:** Min-Jeong Kang, Shin-Young Park, Joong-Soo Han

**Affiliations:** 1 Department of Biomedical Sciences, Graduate School for Biomedical Science and Engineering, Hanyang University, Seoul, Republic of Korea; 2 Biomedical Research Institute, Department of Biochemistry and Molecular Biology, College of Medicine, Hanyang University, Seoul, Republic of Korea

**Keywords:** Hippocalcin, *miR*-*24*-*3p*, Neuronal differentiation, Synaptophysin, SH-SY5Y cells

## Abstract

Hippocalcin (HPCA) is a neuron-specific calcium-binding protein predominantly expressed in the nervous system. In the present study, we demonstrate that HPCA regulates neuronal differentiation in SH-SY5Y cells. We observed that the expression level of HPCA was increased during neuronal differentiation. Depletion of HPCA inhibited both neurite outgrowth and synaptophysin (SYP) expression, whereas overexpression of HPCA enhanced neuronal differentiation. Interestingly, we also found that the expression of *HPCA* mRNA was modulated by *miR*-*24*-*3p*. Using a dual-luciferase assay, we showed that co-transfection of a plasmid containing the *miR*-*24*-*3p* binding site from the 3′-untranslated region (3′UTR) of the *HPCA* gene and an *miR*-*24*-*3p* mimic effectively reduced luminescence activity. This effect was abolished when *miR*-*24*-*3p* seed sequences in the 3′UTR of the *HPCA* gene were mutated. *miR*-*24*-*3p* expression was decreased during differentiation, suggesting that the decreased expression level of *miR*-*24*-*3p* might have upregulated mRNA expression of *HPCA*. As expected, upregulation of *miR*-*24*-*3p* by an miRNA mimic led to reduced HPCA expression, accompanied by diminished neuronal differentiation. In contrast, downregulation of *miR*-*24*-*3p* by an antisense inhibitor promoted neurite outgrowth as well as levels of SYP expression. Taken together, these results suggest that *miR*-*24*-*3p* is an important miRNA that regulates neuronal differentiation by controlling HPCA expression.

**Electronic supplementary material:**

The online version of this article (10.1007/s00018-019-03290-3) contains supplementary material, which is available to authorized users.

## Introduction

MicroRNAs (miRNAs) are small, highly conserved non-coding RNA molecules of approximately 22 nucleotides. They can modulate gene expression through complementary base pairing of the seed sequence (the 2–8 nucleotides from the 5′ end of the miRNA) located in the 3′-untranslated region (3′UTR) of the target mRNA, leading to the translational suppression and/or destabilization of the target mRNAs [1–3]. It is widely accepted that a single miRNA has the potential to inhibit the expression of hundreds of target mRNAs and, conversely, individual mRNAs are commonly targeted by multiple miRNAs [4]. Hence, miRNAs serve as key regulators in various biological processes, such as proliferation, differentiation, apoptosis, metabolism, and development [5–8]. In mammals, many miRNAs are spatially expressed in the brain, suggesting their essential roles in central nervous system development and function [9, 10]. The specific knockdown of Dicer, the key enzyme involved in processing miRNA precursors into mature miRNAs, leads to malformation of the midbrain and cerebellum and failure of neural crest and dopaminergic differentiation [11, 12]. Recent studies have also demonstrated the contribution of individual miRNAs to various aspects of synaptic development and plasticity. *miR*-*132* is involved in activity-dependent structural and functional plasticity by targeting the Rho family GTPase-activating protein, p250GAP [13]. Brain-specific *miR*-*124a* is also critical for the differentiation and maturation of dentate gyrus neurons and retinal cone cells [14]. In addition, *miR*-*335*-*5p* plays a key role in spatial memory formation and hippocampal plasticity [15]. Therefore, miRNAs are key regulators of mammalian neurodevelopment and have been increasingly linked to neuronal differentiation and the translatability of mRNAs in neurons. Our previous study showed that HPCA expression was gradually increased during neuronal differentiation [16]. Therefore, we assumed that an miRNA would be associated with HPCA expression during neuronal differentiation. Using an miRNA Target Prediction algorithm, we found that only *miR*-*24*-*3p* had two potential binding sites to 3′UTR of *HPCA* among several miRNAs. *miR*-*24*-*3p*, which belongs to the *miR*-*24* family, is a well-known cancer-associated miRNA that is differentially expressed in the initiation and progression in multiple kinds of tumors. Inhibition of *miR*-*24*-*3p* promotes osteogenic differentiation by targeting Smad5 [17]. *miR*-*24*-*3p* also facilitates cell proliferation and inhibits cell apoptosis in gastric cancer by targeting BCL2L11 [18]. However, there is no evidence of any association with neurons in *miR*-*24*-*3p*.

Hippocalcin (HPCA) is a high-affinity calcium-binding protein expressed most abundantly in pyramidal cells of the hippocampal CA1 region [19, 20]. HPCA is a member of the neuronal calcium sensor protein family. It has four EF-hand-structured Ca^2+^-binding domains and a myristoylation site in the N-terminus, which allow its translocation from the cytosol to the plasma membrane and/or perinuclear compartments in response to increased Ca^2+^ concentration [21–25]. HPCA acts as a molecular linker between calcium entry into neurons through *N*-methyl-d-aspartate receptors and the regulated endocytosis of α-amino-3-hydroxy-5-methyl-4-isoxazolepropionic acid receptors [26]. During brain development, HPCA expression in hippocampal pyramidal cells abruptly increases, concurrent with synaptic formation [27]. Our previous study showed that HPCA increases NeuroD expression, resulting in neurite outgrowth during the differentiation of H19-7 cells [28]. Moreover, it was found to promote neuronal differentiation through activation of the PKCα/PLD1/SHP1 cascade, leading to the inhibition of astrocytic differentiation in cortical neural stem cells [16]. We also reported that HPCA stimulates astrocytic differentiation through the activation of STAT3 in hippocampal neural precursor cells [29]. However, the role of HPCA regulation by miRNAs in neural differentiation has not previously been studied.

Here, we showed that HPCA has an important role in the neuronal differentiation of SH-SY5Y cells. In addition, we identified *miR*-*24*-*3p* as a putative regulator of HPCA during neuronal differentiation. We observed that overexpression of *miR*-*24*-*3p* reduced *HPCA* mRNA levels and inhibited neuronal differentiation, whereas the inhibition of *miR*-*24*-*3p* had the opposite effects. Taken together, our findings suggest that the modulation of HPCA expression or *miR*-*24*-*3p* function may be used to regulate neuronal differentiation.

## Materials and methods

### Materials

For cell culture experiments, Dulbecco’s modified Eagle medium (DMEM) containing l-glutamine, high glucose concentration, and pyruvate and fetal bovine serum (FBS) were purchased from Gibco (Grand Island, NY, USA) and all-trans retinoic acid (RA) was purchased from Sigma-Aldrich (St Louis, MO, USA). A penicillin/streptomycin solution and trypsin/EDTA were purchased from WelGENE, Inc. (Daegu, Korea). Anti-HPCA (#ab24560) and anti-synaptophysin (SYP, #ab32127) antibodies were purchased from Abcam (Cambridge, UK), an anti-calnexin antibody (#ADI-SPA-860-F) was purchased from Enzo Life Sciences (Farmingdale, NY, USA), and an Alexa Fluor^®^ 488-conjugated secondary goat anti-rabbit IgG (H + L) antibody (#A-11008) was purchased from Invitrogen (Carlsbad, CA, USA). All other chemicals were of analytical grade.

### Cell culture and differentiation conditions

SH-SY5Y and HeLa cells were maintained in DMEM supplemented with 10% heat-inactivated FBS and 1% penicillin/streptomycin and incubated in a humidified atmosphere at 37 °C with 5% CO_2_. The medium was replaced every 2 days and cells were split before they reached confluence. SH-SY5Y cells were differentiated by treatment with 50 μM RA for 5 or 7 days. The culture medium was replaced on alternate days with fresh medium containing 50 μM RA.

### RNA interference

For HPCA silencing experiments, an *HPCA* siRNA (ON-TARGET plus SMARTpool, #L-017429-02-0010) and a negative control siRNA (ON-TARGET plus Non-targeting pool, #D-001810-10-20) were purchased from Dharmacon (Lafayette, CO, USA). Transient siRNA transfections were performed in 12-well plates by introducing 100 nM *HPCA* siRNA or negative control siRNA into SH-SY5Y cells using Lipofectamine RNAiMAX transfection reagent (Invitrogen), according to the manufacturer’s protocol.

### Retrovirus construction and transduction

HPCA cDNA was cloned into a retroviral vector containing IRES-EGFP. Virus particles were produced by transfecting the retrovirus packaging cell line, 293GPG, with the vector using Lipofectamine 3000 transfection reagent (Invitrogen) and harvesting supernatants containing viral particles after incubation for 48 h. For virus transduction, SH-SY5Y cells were incubated with a viral suspension (4 × 10^6^ particles/mL) containing polybrene (1 μg/mL, δ-Aldrich) for 4 h, followed by transfer to fresh culture medium containing 50 μM RA.

### Transfection of miRNA mimic and inhibitor

The *miR*-*24*-*3p* mimic and negative control miRNA were synthesized by Genolution (Seoul, Korea). A sequence-specific *miR*-*24*-*3p* inhibitor (cat. no. IH-300497-05-0010, miRIDIAN microRNA Human hsa-*miR*-*24*-*3p*—Hairpin Inhibitor) and its corresponding non-specific control (cat. no. IN-001005-01-20, miRIDIAN microRNA Hairpin Inhibitor Negative Control #1) were purchased from Dharmacon. The sequences of the *miR*-*24*-*3p* mimic and inhibitor are provided in Supplementary Table 1. HeLa or SH-SY5Y cells were seeded 1 day before transfection. Each mimic or inhibitor was transiently transfected into cells using Lipofectamine RNAiMAX transfection reagent, as described in the manufacturer’s instructions.

### RNA extraction and reverse transcription-quantitative polymerase chain reaction (RT-qPCR)

Total RNA was extracted from cultured cells using RNAiso Plus (Takara Bio Inc., Ohtsu, Japan). cDNA was prepared for qPCR by reverse transcribing 300 ng of purified total RNA using GoScript™ Reverse Transcriptase and random primers (Promega Corporation, Madison, WI, USA). qPCR was performed using a SensiFAST™ SYBR No-ROX Kit (Bioline, London, UK) on a CFX Connect™ Real-Time PCR Detection System (Bio-Rad, Hercules, CA, USA). The primers used for RT-qPCR are listed in Supplementary Table 1. Thermocycling conditions were 95 °C for 10 min, followed by 40 cycles of 95 °C for 15 s and 60 °C for 1 min. Each sample was tested in duplicate and at least three samples obtained from independent experiments were analyzed. Relative quantification was carried out using the 2^−ΔΔCt^ method. Gene expression was normalized to the internal control, *GAPDH*.

## Western blotting assays

Cells were lysed in ice-cold hypotonic lysis buffer consisting of 10 mM Tris–HCl (pH 7.5), 10 mM NaCl, 10 mM EDTA, 0.5% Triton X-100, and Complete™ EDTA-free Protease Inhibitor Cocktail (Roche Diagnostics, Indianapolis, IN, USA). Protein samples (15–30 μg) were electrophoresed in NEXT GEL^®^ 15% polyacrylamide gels (Amresco, Solon, OH, USA) and then transferred to PVDF membranes (Merck Millipore, Darmstadt, Germany). After blocking with 5% non-fat dried milk for 1 h, the membranes were incubated with rabbit anti-HPCA (1:500 dilution), rabbit anti-SYP (1:20,000 dilution), and rabbit anti-calnexin (1:2000 dilution) primary antibodies, followed by an HRP-conjugated secondary antibody (1:2000; Jackson ImmunoResearch, West Grove, PA, USA). Specific bands were detected by enhanced chemiluminescence (Thermo Fisher Scientific, Rockford, IL, USA) and were quantified using Image J software (v1.51 k, NIH, http://www.rsb.Info.nih.gov/ij/).

### Immunofluorescence staining

Cells were initially fixed with 4% (w/v) paraformaldehyde in phosphate-buffered saline (PBS) for 20 min and then washed three times with 0.1% BSA in PBS at room temperature. After blocking with 10% normal goat serum in 0.1% BSA in PBS containing 0.3% Triton X-100 for 1 h at room temperature, cells were immunostained with a rabbit monoclonal anti-SYP primary antibody (1:200 dilution) at 4 °C overnight. Subsequently, cells were washed three times with PBS and then labeled with a 1:500 dilution of an Alexa Fluor^®^ 488-conjugated goat anti-rabbit IgG (H + L) secondary antibody for 1 h, before mounting with Vectashield mounting medium (Vector Laboratories, Burlingame, CA, USA) containing 4,6-diamidino-2-phenylindole (DAPI). Immunoreactive cells were analyzed under an epifluorescence microscope (Nikon Instruments, Melville, NY, USA) at magnifications ranging from 20× to 40×.

### Measurement of neurite outgrowth

Cells were cultured on coverslips in 24-well plates, fixed with 0.1% (w/v) picric acid/PBS containing 4% (w/v) paraformaldehyde, and incubated overnight at 4 °C with an anti-SYP antibody (1:200). After incubation with a 1:2000 dilution of Alexa Fluor^®^ 488-conjugated goat anti-rabbit IgG secondary antibody, cells were mounted on slides with Vectashield. SYP-positive cells were photographed using an epifluorescence microscope. Neurite outgrowth was assessed by measuring the length of neurites in all positively identified neurite-bearing cells. The mean neurite length per cell was calculated using Image J software. The length of the primary neurite was defined as the distance from the soma to the tip of the branch. For this analysis, a cell was arbitrarily defined as neurite bearing when it bore at least one process equal to or longer than 25 μm (mean diameter of the cell body) [30]. For each graph, neurite length data were generated from randomly selected areas of at least five independent cultures from three independent experiments, and more than 100 cells were counted for each condition in each experiment.

### Plasmid construction and dual-luciferase reporter assays

miRNA target sequences in the 3′UTR of the *hHPCA* gene were inserted using the XbaI restriction site in the pmirGLO vector (Promega Corporation, Cat. #E1330). The sequences targeted by *miR*-*24*-*3p* are summarized in Supplementary Table 1. The correct orientation and nucleotide sequence of the 3′UTR fragments in the plasmid constructs were further confirmed by sequencing analysis.

Dual-luciferase reporter plasmids were co-transfected with miRNA mimics or miRNA inhibitors into HeLa cells and after 48 h, cells were assayed for luciferase activity using the Dual-Glo^®^ Luciferase Assay System (Promega Corporation, Cat. #E2920) and a luminometer (Promega Corporation), according to the manufacturer’s instructions. To correct for variations in transfection efficiency, firefly luciferase activity was normalized to *Renilla* luciferase activity. Firefly luciferase activity was calculated as the percent activity relative to luciferase activity in cells transfected with negative control miRNA mimics or inhibitors. For each transfection, luciferase activity was averaged from at least three replicates.

### TaqMan RT-qPCR of miRNA

For the detection of endogenous miRNA expression, 10 ng of purified total RNA was reverse transcribed using a TaqMan^®^ MicroRNA Reverse Transcription Kit (Applied Biosystems, Foster City, CA, USA). The resulting cDNA was used amplified by PCR using a TaqMan^®^ MicroRNA Assay (Applied Biosystems) according to the manufacturer’s instructions. cDNA amplification and detection of products were performed using a CFX Connect™ Real-Time PCR Detection System, with an initial denaturation at 95 °C for 10 min, followed by 40 cycles of amplification at 95 °C for 15 s and 60 °C for 1 min, before cooling. The threshold cycle (Ct) for *miR*-*24*-*3p* was automatically defined within the linear amplification phase and was normalized to the control, *U6* snRNA, to calculate the ΔCt value. The relative difference in *miR*-*24*-*3p* expression levels (ΔΔCt) in the sorted cells was calculated and presented as fold induction (2^−ΔΔCt^).

### Statistical analysis

All quantitative data are expressed as mean ± standard error of the mean (SEM). In each experiment, all measurements were performed at least in triplicate. Data were analyzed using a two-tailed, unpaired Student’s *t* test and values of *P* < 0.05 were considered statistically significant.

## Results

### HPCA was upregulated during the neuronal differentiation of SH-SY5Y cells

**Fig. 1:**
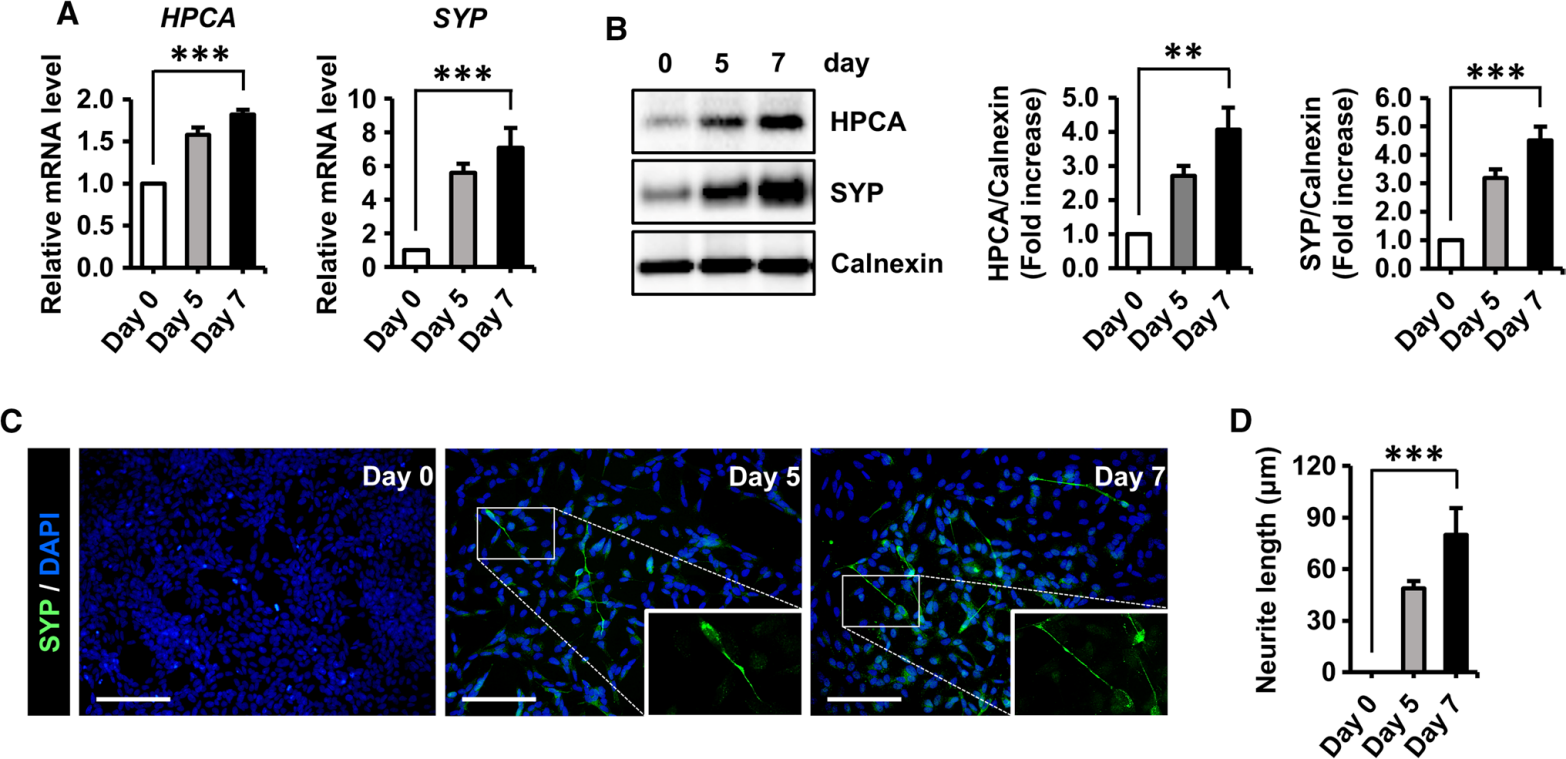
Differentiation induced expression of HPCA in SH-SY5Y cells. **a**, **b** SH-SY5Y cells were induced to differentiate by adding 50 μM all-trans retinoic acid (RA) for the indicated number of days. **a** The mRNA levels of the presynaptic marker, synaptophysin (*SYP*) and hippocalcin (*HPCA*), were determined by RT-qPCR. **b** Proteins were analyzed by western blotting with anti-HPCA, anti-SYP, and anti-calnexin antibodies. The graph shows mean densities as fold increases from five independent experiments (mean ± SEM). Band intensities were quantified using Quantity One^®^ software. **P < 0.01, ***P < 0.001 compared with day 0 of differentiation. **c** Neuronal differentiation was induced by adding 50 μM RA for the indicated number of days. Cells were stained with DAPI (blue) and an anti-SYP antibody (green) to visualize neurite extensions. Scale bar, 100 μm. **d** Neurite lengths were measured in randomly selected areas in three independent experiments. ***P < 0.001 compared with day 0 of differentiation (mean ± SEM)

The human neuroblastoma cell line, SH-SY5Y, has been widely used as an in vitro model for neuroscience research because these cells are easily induced to differentiate by RA treatment [31–33]. To determine the involvement of HPCA in regulating human neuronal differentiation, SH-SY5Y cells were induced to differentiate in the presence of 50 μM RA for 5 or 7 days. As shown in Fig. 1a and b, the mRNA and protein levels of HPCA and SYP, a presynaptic neuronal marker, were gradually increased under differentiation conditions. Immunostaining showed a significant increase in the number of SYP-positive cells and neurite outgrowth (0 μm versus 79.9 ± 15.5 μm, *P* < 0.001, Fig. 1c, d) during differentiation. These data suggest that HPCA is involved in the neuronal differentiation of SH-SY5Y cells.

### HPCA regulated the differentiation of SH-SY5Y cells

**Fig. 2:**
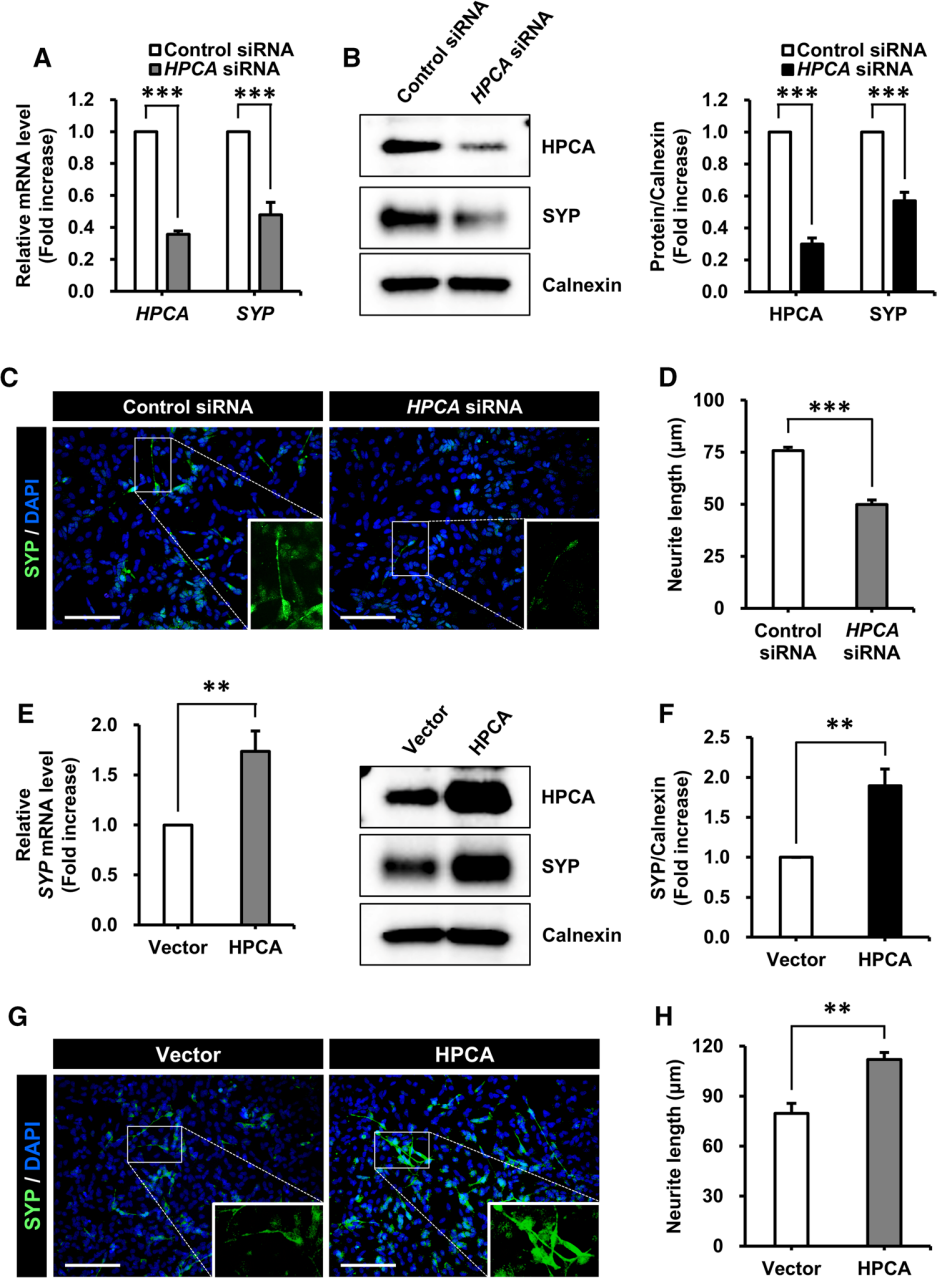
Effect of HPCA on the neuronal differentiation of SH-SY5Y cells. **a**, **b** SH-SY5Y cells were transiently transfected with control siRNA or *HPCA* siRNA, and then incubated for 7 days after the addition of RA. **a***HPCA* and *SYP* mRNA levels were analyzed by RT-qPCR. **b** HPCA, SYP, and calnexin protein levels were determined by western blotting. The graph shows mean densities as fold increases from four independent experiments (mean ± SEM). Band intensities were quantified using Quantity One^®^ software. ***P < 0.001 compared with the day 0 of differentiation. **c** Cells were transfected with control siRNA or *HPCA* siRNA and then induced to differentiate by adding RA. After 7 days, fixed cells were stained with an anti-SYP antibody (green) and DAPI (blue). Scale bar, 100 μm. **d** Neurite lengths were measured in randomly selected areas from five independent cultures. ***P < 0.001 compared with the control siRNA (mean ± SEM). **e**, **f** SH-SY5Y cells were transduced with pMSCV-IRES-EGFP or pMSCV-HPCA-Myc-IRES-EGFP and induced to differentiate for 7 days. **e***SYP* mRNA levels were detected by RT-qPCR. **f** Cells were lysed and analyzed by western blotting with anti-HPCA, anti-SYP, and anti-calnexin antibodies. The graph shows mean densities as fold increases from five independent experiments (mean ± SEM). Band intensities were quantified with Quantity One^®^ software. **P < 0.01 compared with day 0 of differentiation. **g** Immunofluorescence was used to visualize SYP (green) at day 7 of differentiation, with or without overexpression of HPCA. Nuclei were stained with DAPI (blue). Scale bar, 100 μm. **h** Neurite lengths were measured in randomly selected areas from five slides of each condition. **P < 0.01 compared with the vector control (mean ± SEM)

Previously, we reported that HPCA is critical for neuronal differentiation and neurite outgrowth in multiple cell types [16, 28]. To investigate whether HPCA was implicated in neuronal differentiation, either control siRNA or *HPCA* siRNA were transiently transfected into SH-SY5Y cells, which were then allowed to differentiate for 7 days. We showed that mRNA and protein levels of SYP were markedly attenuated by HPCA knockdown (Fig. 2a, b). To determine the role of HPCA in neuronal differentiation, we measured neurite length after 7 days of differentiation in the presence of *HPCA* siRNA. *HPCA* siRNA transfection significantly decreased neurite outgrowth (75.7 ± 1.6 μm versus 49.9 ± 2.2 μm, *P* < 0.001, Fig. 2c, d) when compared with control siRNA transfection, thus implicating HPCA in the regulation of SYP expression and neurite outgrowth during differentiation.

To confirm the role of HPCA in neuronal differentiation, cells were transduced with pMSCV-IRES-EGFP or pMSCV-HPCA-MYC-IRES-EGFP and induced to differentiate for 7 days. As shown in Fig. 2e, f, SYP mRNA and protein expression levels were significantly increased by HPCA overexpression. Moreover, HPCA markedly increased neurite outgrowth (79.7 ± 6.0 μm versus 112.0 ± 4.2 μm, *P* < 0.01, Fig. 2g, h) compared with the vector control. These findings suggest that HPCA is required for the neuronal differentiation of SH-SY5Y cells.

### *miRNA*-*24*-*3p* targeted endogenous HPCA expression

**Fig. 3:**
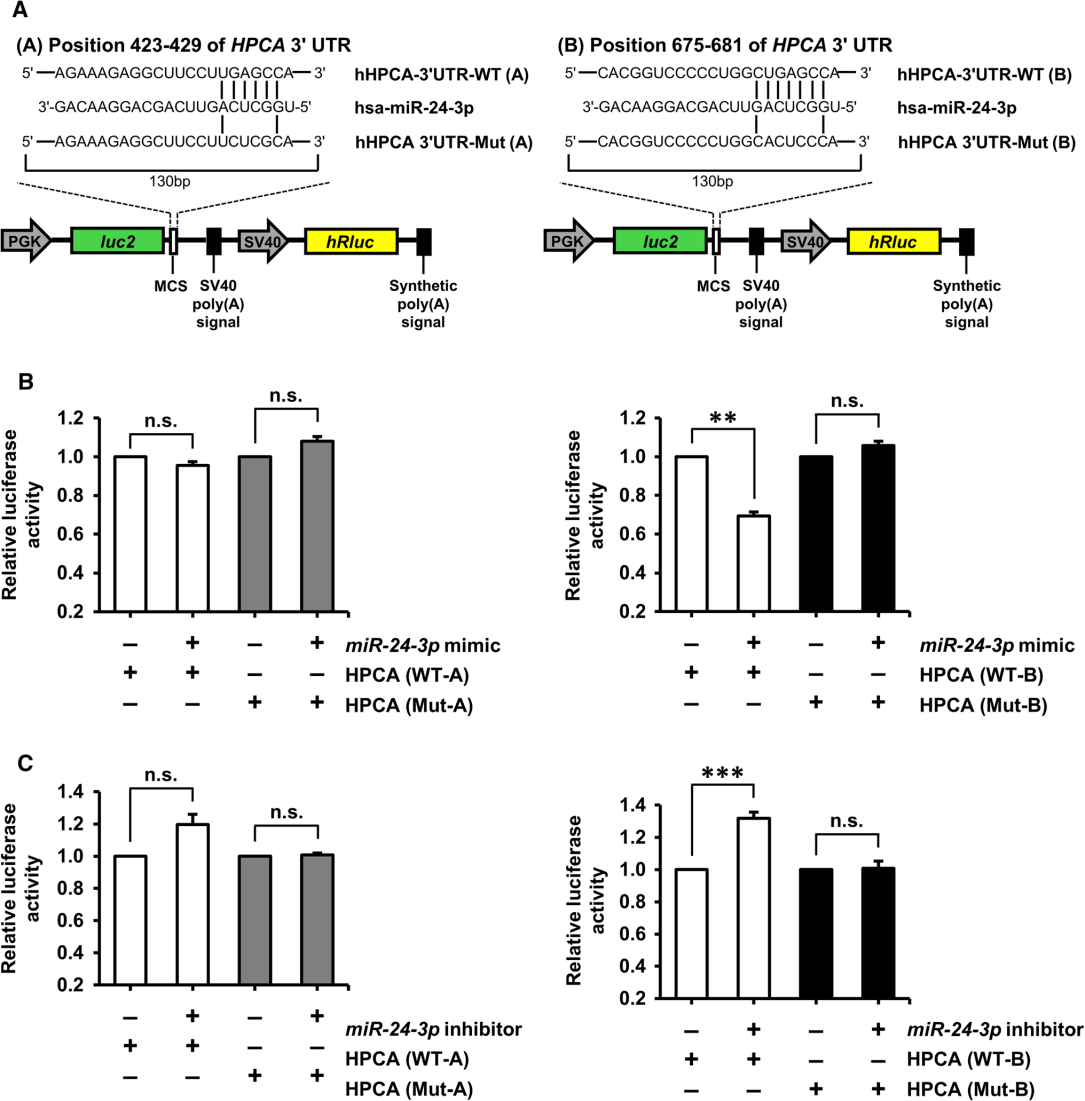
Validation of *HPCA* as a direct target of *miR*-*24*-*3p.***a** Map of representative bicistronic firefly/*Renilla* luciferase (FLuc/RLuc) plasmids containing *HPCA* mRNA 3′UTRs with putative *miR*-*24*-*3p* binding sites (WT) or mutated binding sites (Mut). **b** A control miRNA mimic or an *miR*-*24*-*3p* mimic was co-transfected with HPCA-3′UTR-WT or HPCA-3′UTR-Mut into HeLa cells. **c** A control miRNA inhibitor or an *miR*-*24*-*3p* inhibitor was co-transfected with HPCA-3′UTR-WT or HPCA-3′UTR-Mut into HeLa cells. Luciferase assays were performed to quantify the relative luciferase activity of FLuc normalized to the activity of RLuc. The relative activity of luciferase in control miRNA inhibitor-transfected cells was set to 1.0. **P < 0.01; ***P < 0.001; *n.s.* not significant

In the present study, we showed that HPCA expression was increased with the induction of differentiation. An important mode of post-transcriptional regulation is miRNA-mediated repression of mRNA transcripts. To identify miRNAs that target *HPCA* mRNA, we performed *in silico* screening using TargetScan (http://www.targetscan.org). The TargetScan database predicted two putative *miR*-*24*-*3p* binding sites in the 3′UTR of *HPCA* (positions 423–429 and 675–681 of *HPCA* 3′UTR, Fig. 3a), which suggested that *miR*-*24*-*3p* may serve as a suppressor of *HPCA* mRNA expression. To confirm that *miR*-*24*-*3p* bound to the *HPCA* mRNA 3′UTR, we constructed wild-type (WT-A and B) and mutant (Mut-A and B) plasmids encoding either the full or partial sequence of the 3′UTR of *HPCA*, including the predicted *miR*-*24*-*3p* target sites, and performed a luciferase reporter assay (Fig. 3a). Transfection with the *miR*-*24*-*3p* mimic specifically decreased the activity of a luciferase reporter gene fused to HPCA-3′UTR-WT-B (position 675–681: *P* < 0.01), but had no effect on HPCA-3′UTR-WT-A (position 423–429, Fig. 3b). The activity of the reporter gene fused to HPCA-3′UTR-Mut-B was unaffected by the presence of exogenous *miR*-*24*-*3p* (Fig. 3b).

**Fig. 4:**
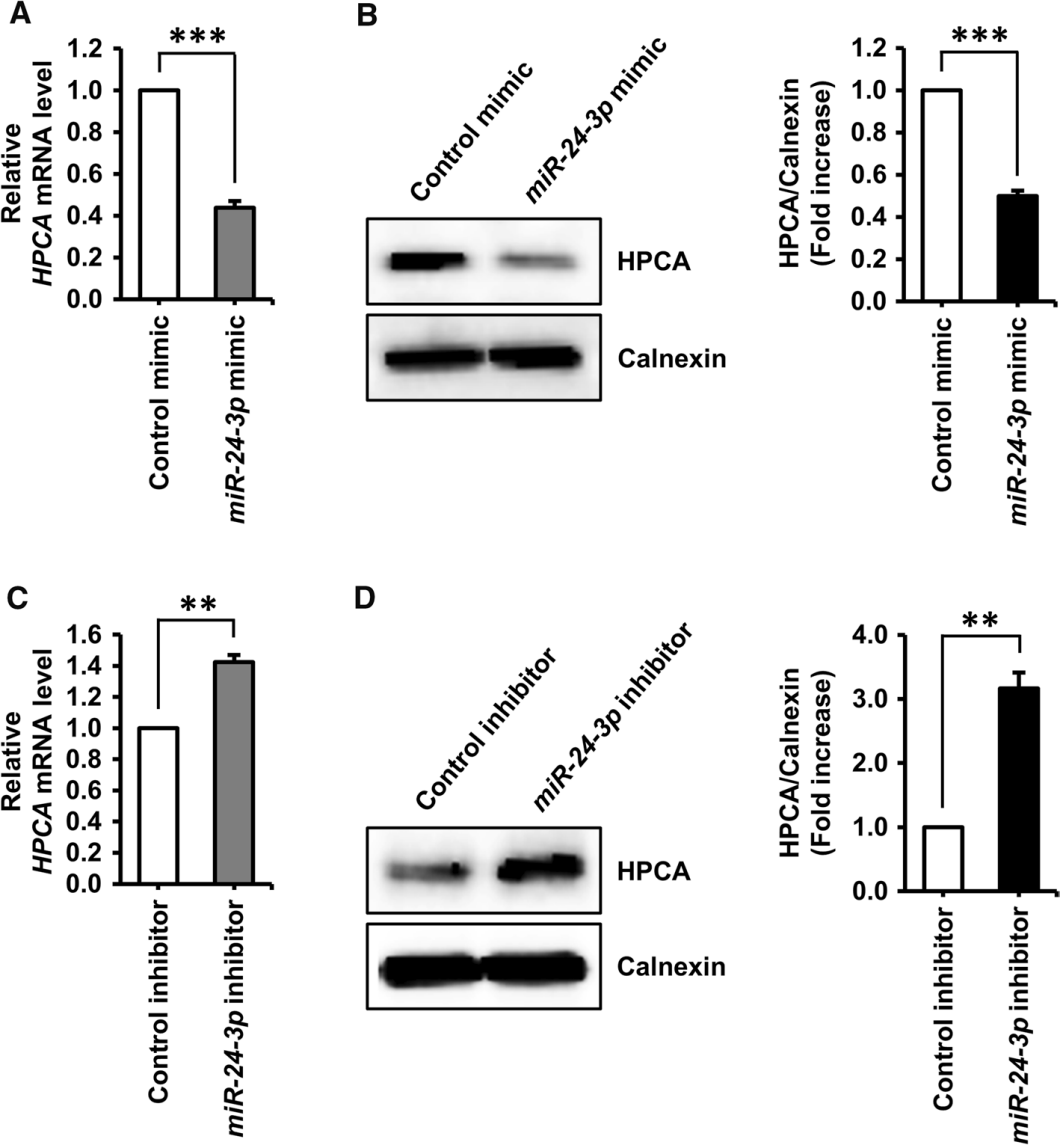
Identification of *miR*-*24*-*3p* as a potential upstream regulator of HPCA. **a**, **b** HeLa cells were transiently transfected with a control miRNA mimic or an *miR*-*24*-*3p* mimic for 2 days. **a***HPCA* mRNA levels were detected by RT-qPCR. **b** Cells were lysed and analyzed by western blotting with anti-HPCA and anti-calnexin antibodies. The graph shows mean densities as fold increases from three independent experiments (mean ± SEM). Band intensities were quantified using Quantity One^®^ software. ***P < 0.001 compared with the control miRNA mimic. **c**, **d** Cells were transfected with a control miRNA inhibitor or an *miR*-*24*-*3p* inhibitor for 3 days. **c***HPCA* mRNA levels were analyzed by RT-qPCR. **d** Proteins were analyzed by western blotting with anti-HPCA and anti-calnexin antibodies. The graph shows mean densities as fold increases from three independent experiments (mean ± SEM). Band intensities were quantified with Quantity One^®^ software. **P < 0.01 compared with the control miRNA inhibitor

Next, we performed additional experiments using a specific inhibitor of *miR*-*24*-*3p*. Co-transfection of HPCA-3′UTR-WT-B with an *miR*-*24*-*3p*-specific inhibitor caused a significant increase (approximately 40%, *P* < 0.001) in luciferase activity, compared with the control inhibitor. Luciferase activity was not affected when HPCA-3′UTR-WT-A was co-transfected with an *miR*-*24*-*3p*-specific inhibitor (Fig. 3c). These results demonstrated that position 675–681 of the human *HPCA* 3′UTR is a functional binding site for *miR*-*24*-*3p*. Furthermore, we confirmed that *miR*-*24*-*3p* regulated endogenous HPCA expression. As shown in Fig. 4a, b, an *miR*-*24*-*3p* mimic decreased HPCA mRNA and protein levels, compared with a control mimic. By contrast, an *miR*-*24*-*3p* inhibitor significantly increased the levels of HPCA mRNA and protein (Fig. 4c, d). Taken together, these data indicated that *miR*-*24*-*3p* inhibited HPCA expression by binding to a single site present in the *HPCA* mRNA 3′UTR.

### *miR*-*24*-*3p* modulated neuronal differentiation by inhibiting HPCA in SH-SY5Y cells

**Fig. 5:**
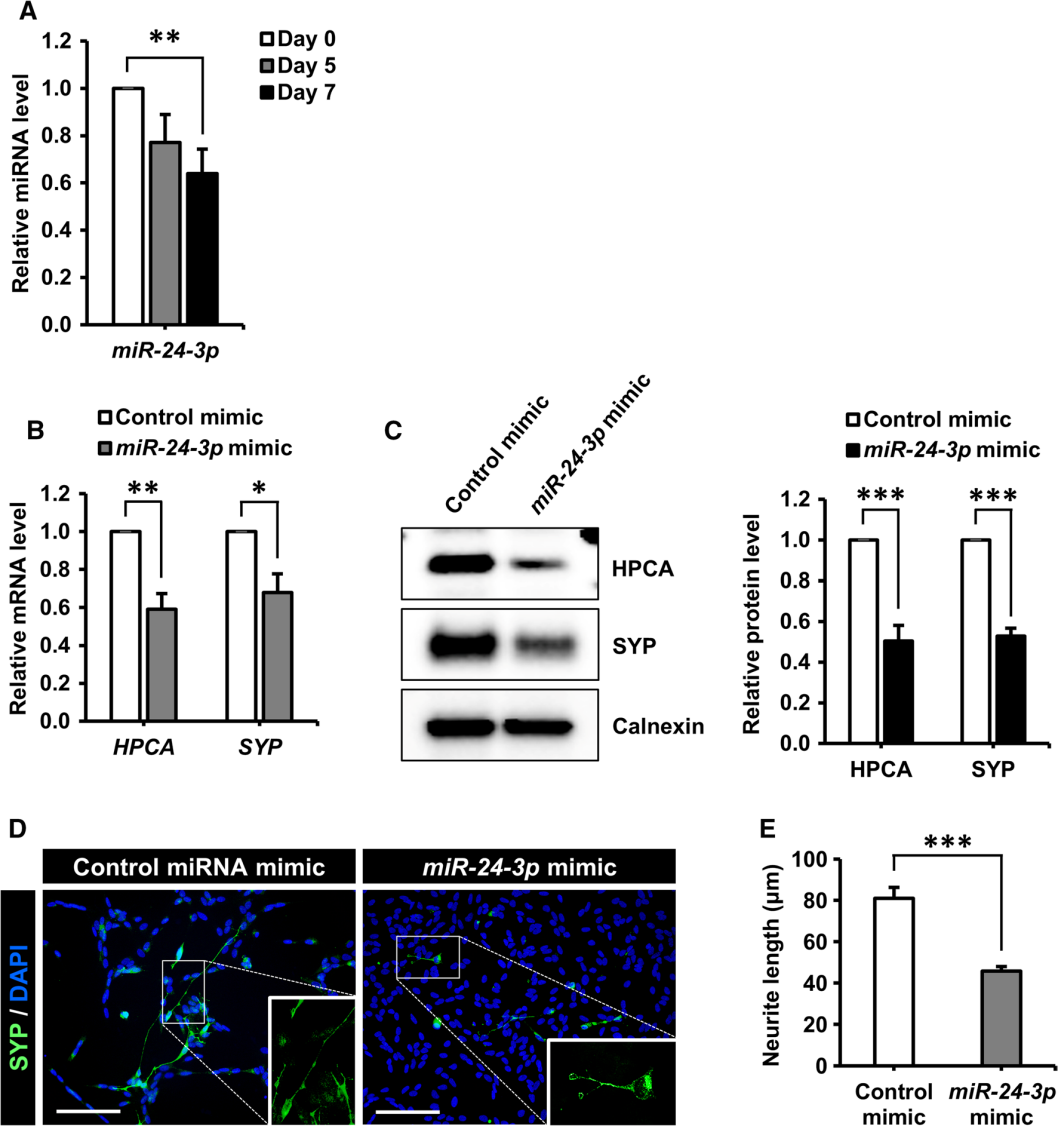
*miR*-*24*-*3p* regulates neuronal differentiation, presumably by reducing HPCA expression in SH-SY5Y cells. **a** Mature *miR*-*24*-*3p* levels during the differentiation of SH-SY5Y cells were quantified using TaqMan RT-qPCR. **P < 0.01 compared with day 0 of differentiation (mean ± SEM; *n* = 4). **b**, **c** Differentiation was induced in SH-SY5Y cells transfected with a control miRNA mimic or an *miR*-*24*-*3p* mimic. **b***HPCA* and *SYP* mRNA levels were analyzed by RT-qPCR. **c** HPCA, SYP, and calnexin protein levels were determined by western blotting. The graph shows mean densities as fold increases from four independent experiments (mean ± SEM). Band intensities were quantified using Quantity One^®^ software. *P < 0.05, **P < 0.01, ***P < 0.001 compared with the control miRNA mimic at day 7 of differentiation. **d** Cells were transfected with a control miRNA mimic or an *miR*-*24*-*3p* mimic and induced to differentiate for 7 days. Fixed cells were stained with an anti-SYP antibody (green) and DAPI (blue). Scale bar, 100 μm. **e** Neurite lengths were measured in randomly selected areas from three experiments (mean ± SEM). ***P < 0.001 compared with the control miRNA mimic at day 7 of differentiation

**Fig. 6:**
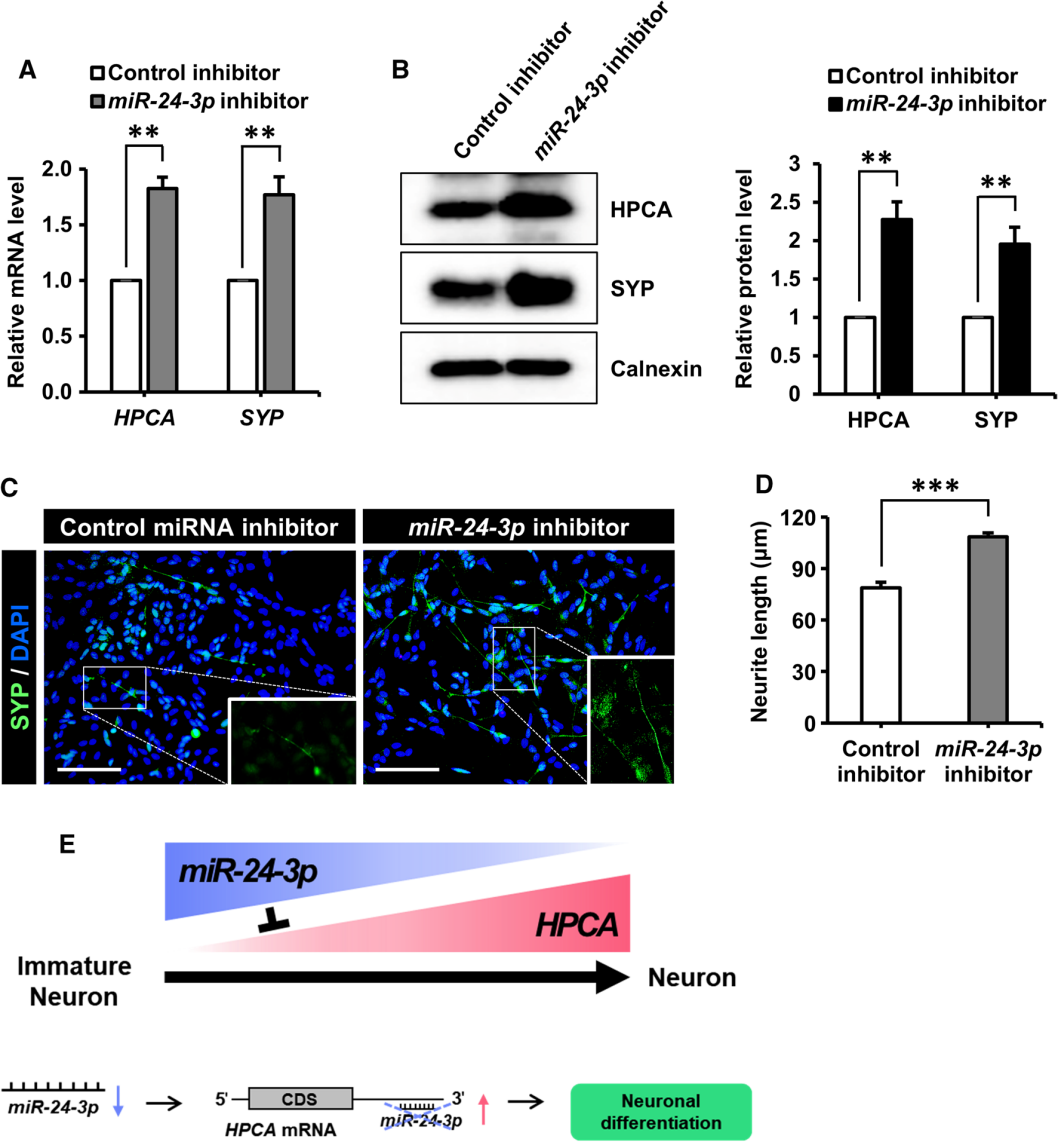
Inhibition of *miR*-*24*-*3p* promoted the neuronal differentiation of SH-SY5Y cells. **a**, **b** SH-SY5Y cells at day 2 of differentiation were transfected with a control miRNA inhibitor or an *miR*-*24*-*3p* inhibitor and were allowed to differentiate for 5 days. **a***HPCA* and *SYP* mRNA levels during the differentiation period were detected by RT-qPCR. **b** Cells were lysed and analyzed by western blotting with anti-HPCA, anti-SYP, and anti-calnexin antibodies. The graph shows mean densities as fold increases from four independent experiments (mean ± SEM). Band intensities were quantified using Quantity One^®^ software. **P < 0.01 compared with the control miRNA inhibitor at day 7 of differentiation. **c** Immunofluorescence was used to visualize SYP (green) at day 7 of differentiation with a control miRNA inhibitor or an *miR*-*24*-*3p* inhibitor. Nuclei were stained with DAPI (blue). Scale bar, 100 μm. **d** Neurite lengths were measured in random areas from five cultures (mean ± SEM). ***P < 0.001 compared with the control miRNA inhibitor. **e** The schematic model suggests that the inhibition of *miR*-*24*-*3p* promotes neuronal differentiation by increasing HPCA expression levels in SH-SY5Y cells

These findings prompted us to test whether the downregulation of *miR*-*24*-*3p* was responsible for the increased level of HPCA expression during neuronal differentiation. SH-SY5Y cells were differentiated and *miR*-*24*-*3p* levels were quantified by TaqMan qPCR assay (Fig. 5a). *miR*-*24*-*3p* levels were downregulated by approximately 40%, suggesting that decreased *miR*-*24*-*3p* expression may have been responsible for the increase in *HPCA* mRNA levels. To elucidate the role of *miR*-*24*-*3p* in the neurogenesis of SH-SY5Y cells, we transfected cells with an *miR*-*24*-*3p* mimic or a control mimic and then induced neuronal differentiation by RA treatment for 7 days. The *miR*-*24*-*3p* mimic suppressed SYP expression, as well as HPCA mRNA (Fig. 5b) and protein (Fig. 5c) levels. Moreover, the *miR*-*24*-*3p* mimic significantly decreased neurite outgrowth (81.0 ± 5.3 μm versus 45.7 ± 2.3 μm, *P* < 0.001, Fig. 5d, e) compared with the control mimic, indicating that *miR*-*24*-*3p* may regulate neuronal differentiation by targeting HPCA in SH-SY5Y cells. To further confirm the role of *miR*-*24*-*3p* in the neuronal differentiation of SH-SY5Y cells, we transfected cells with an *miR*-*24*-*3p* inhibitor or a control inhibitor. As expected, *miR*-*24*-*3p* inhibition promoted the levels of SYP and HPCA, when compared with the control inhibitor (Fig. 6a, b). Moreover, the *miR*-*24*-*3p* inhibitor significantly increased neurite outgrowth (78.7 ± 3.1 μm versus 108.4 ± 2.3 μm, *P* < 0.001, Fig. 6c, d) during the neuronal differentiation of SH-SY5Y cells. Taken together, these results demonstrated that *miR*-*24*-*3p* modulated neuronal differentiation by targeting HPCA expression in SH-SY5Y cells.

## Discussion

HPCA has multiple functions in various cellular processes, including Ca^2+^ buffering, neuroprotection, and receptor-mediated endocytosis, by detecting and binding calcium to increase intracellular calcium influx [25, 26, 34]. We have previously shown that HPCA promotes NeuroD expression, resulting in neurite outgrowth during differentiation of H19-7 cells [28]. Furthermore, our previous study demonstrated that HPCA promotes neurogenesis by regulating the PKCα/PKD1/PLD1/SHP-1/STAT3 pathway in neural stem cells [16]. However, the molecular mechanisms involved in the regulation of HPCA expression during neuronal differentiation have not been studied yet. In the present study, we demonstrated a novel mechanism whereby HPCA expression is regulated by *miR*-*24*-*3p* in SH-SY5Y cells.

We observed that HPCA levels were markedly increased during the neuronal differentiation of SH-SY5Y cells. HPCA depletion significantly decreased neurite outgrowth and the expression of the neuronal marker, SYP, whereas overexpression of HPCA increased neurite outgrowth and SYP expression. These results strongly indicate that HPCA is required for the neuronal differentiation of SH-SY5Y cells. This is supported by a previous study showing that HPCA plays an important role in neuronal differentiation in various cell types, including H19-7 cells [28] and cortical neural stem cells [16].

miRNAs are key regulators of gene expression through the translational repression and/or degradation of target transcripts [35–37]. By focusing on miRNA-dependent gene regulation, we integrated bioinformatics-based predictions and found that the 3′UTR of *HPCA* contained two putative *miR*-*24*-*3p* binding sites. miRNAs partially bind to homologous sequences in the 3′UTR of their target genes [38, 39]. Using a dual-luciferase reporter system, we demonstrated that only the predicted *miR*-*24*-*3p* binding site at position 675-681 within the *HPCA* 3′UTR is a target for *miR*-*24*-*3p*. HPCA expression was downregulated by *miR*-*24*-*3p* at the mRNA and protein levels. However, it is not clear how *miR*-*24*-*3p* affects the gene expression levels of HPCA. Therefore, further studies will be focused on identifying mechanisms by which *miR*-*24*-*3p* regulates mRNA degradation and/or translation inhibition of *HPCA.*

Thus far, most research on *miR*-*24*-*3p* has focused on its function in cancer using in vitro model systems. For instance, up or downregulation of *miR*-*24* is important for regulation of the growth and/or apoptosis of various cancer types, including breast cancer [40], lung adenocarcinoma [41], hepatocellular carcinoma [42], and colon cancer [43]. In the present study, we investigated its function in neuronal differentiation. *miR*-*24*-*3p* was found to modulate HPCA-mediated neurite outgrowth and neuronal marker expression in SH-SY5Y cells. However, since miR-24-3p mimics did not completely inhibit the expression of HPCA mRNA and protein levels, the possibility of other miRNAs involved in HPCA-mediated neuronal differentiation cannot be ruled out. Therefore, additional experiments are needed to identify HPCA target miRNAs and this will provide a more complete picture of HPCA regulation by miRNAs during neural development.

In conclusion, our results indicated that *miR*-*24*-*3p* directly targeted HPCA to regulate the neuronal differentiation of SH-SY5Y cells. To the best of our knowledge, this is the first demonstration of *miR*-*24*-*3p* inhibition promoting neuronal differentiation by increasing the expression levels of HPCA. Therefore, our findings suggested that *miR*-*24*-*3p* controlled neuronal differentiation by modulating HPCA expression and implicated the *miR*-*24*-*3p*-mediated regulation of HPCA expression as a potential therapeutic target for neuron development disorders in humans. The findings of the present study are summarized in Fig. 6e.

### Electronic supplementary material

Below is the link to the electronic supplementary material.

Supplementary material 1 (DOCX 31 kb)
