# Supplementary material for: MicroRNA-24-3p regulates neuronal differentiation by controlling hippocalcin expression
Source: Cell Mol Life Sci. 2019 Sep 5;76(22):4569–80. doi: 10.1007/s00018-019-03290-3 (PMC6841749; doi:10.1007/s00018-019-03290-3)
Supplement: Supplementary file 1 — Supplementary material 1 (DOCX 31 kb) [file 18_2019_3290_MOESM1_ESM.docx]

**Supplementary Table S1. Sequences of primers and miRNAs used in this study**

| **RT-qPCR** **primer sequences** | |
| --- | --- |
| **Primer name** | **Sequence** |
| HPCA-F | 5'-GTGGGCCTTCAGCATGTAT-3' |
| HPCA-R | 5'-CATCACGGACGAAACCATCT-3’ |
| SYP-F | 5'-CAACACCTCGGTGGTGTTCG-3' |
| SYP-R | 5'-CCTGAGGCCCGTAGGAATC-3' |
| GAPDH-F | 5'-CAAGATCATCAGCAATGCC-3' |
| GAPDH-R | 5'-CTGTGGTCATGAGTCCTTCC-3' |

| **Mimic miRNA sequences** | |
| --- | --- |
| Mimic negative control | 5'-UUGUACUACACAAAAGUACUGUU-3’ |
| *miR-24-3p* mimic | 5'-UCGCCUAAUGGUCUCUGAGCCAUU-3’ |

| **Cloning primer sequences** | | |
| --- | --- | --- |
| **Primer name** | **Sequence** | **Reference** |
| HPCA_3′UTR_  middle-WT (A) | 5'-GAGTTGGAGAAAGAGGCT  TCCTTGAGCCACCCTTCCCAC  CCCAGCCCTTG-3' | underlined sequence represents the *miR-24-3p* target site |
| HPCA_3′UTR_  middle-Mutant (A) | 5'-GAGTTGGAGAAAGAGGCT  TCCTTCTCGCACCCTTCCCAC  CCCAGCCCTTG-3' |  |
| HPCA_3′UTR_  middle-WT (B) | 5'-AAAGAGACACGGTCCCCC  TGGCTGAGCCCCTGTCCTCCC  CTCTGTCCCCC-3' | underlined sequence represents the *miR-24-3p* target site |
| HPCA_3′UTR_  middle-Mutant (B) | 5'-AAAGAGACACGGTCCCCC  TGGCACTCCCCCTGTCCTCCC  CTCTGTCCCCC-3' |  |
